# Supplementary material for: Global air pollution exposure and poverty
Source: Nat Commun. 2023 Jul 22;14:4432. doi: 10.1038/s41467-023-39797-4 (PMC10363163; doi:10.1038/s41467-023-39797-4)
Supplement: Supplementary file 1 — Supplementary Information [file 41467_2023_39797_MOESM1_ESM.docx]

*Supplementary material*:

Global Air Pollution Exposure and Poverty

# 1. Country-level estimates

**Supplementary** **Table 1. Summary statistics for 211 countries and territories**

|  |  |  | **Safe**  **(< 5** **μg/m^3^)** | | **Unsafe**  **(> 5** **μg/m^3^)** | | **Hazardous**  **(> 35** **μg/m^3^)** | | **Poor ($1.9) and unsafe (> 5** **μg/m^3^)** | | **Poor ($3.20) and unsafe**  **(> 5** **μg/m^3^)** | | **Poor ($5.50) and unsafe**  **(>5** **μg/m^3^)** | | **Poor ($5.50) and hazardous**  **(> 35** **μg/m^3^)** | |
| --- | --- | --- | --- | --- | --- | --- | --- | --- | --- | --- | --- | --- | --- | --- | --- | --- |
|  | **Economy/**  **territory** | **Pop. (m)** | **(m)** | **(%)** | **(m)** | **(%)** | **(m)** | **(%)** | **(m)** | **(%)** | **(m)** | **(%)** | **(m)** | **(%)** | **(m)** | **(%)** |
| 1 | China | 1,431.97 | 18.54 | 1.3 | 1,413.43 | 98.7 | 764.9 | 53.4 | 21.3 | 1.5 |  |  |  |  |  |  |
| 2 | India | 1,377.06 | 17.22 | 1.3 | 1,359.85 | 98.8 | 1,326.6 | 96.3 | 202.5 | 14.7 | 716.8 | 52.1 | 1,135.1 | 82.4 | 1,109.2 | 80.6 |
| 3 | United States | 329.86 | 24.98 | 7.6 | 304.88 | 92.4 | 0.0 | 0.0 | 3.0 | 0.9 | 3.8 | 1.1 | 5.3 | 1.6 | 0.0 | 0.0 |
| 4 | Indonesia | 269.68 | 18.28 | 6.8 | 251.40 | 93.2 | 84.1 | 31.2 | 9.0 | 3.4 | 53.8 | 19.9 | 133.2 | 49.4 | 41.9 | 15.5 |
| 5 | Pakistan | 220.52 | 1.87 | 0.8 | 218.66 | 99.2 | 205.2 | 93.1 | 3.3 | 1.5 | 50.5 | 22.9 | 139.2 | 63.1 | 130.6 | 59.2 |
| 6 | Nigeria | 205.63 | 2.47 | 1.2 | 203.16 | 98.8 | 116.9 | 56.9 | 79.7 | 38.7 | 141.0 | 68.6 | 181.4 | 88.2 | 103.3 | 50.3 |
| 7 | Bangladesh | 162.83 | 3.42 | 2.1 | 159.41 | 97.9 | 159.4 | 97.9 | 12.4 | 7.6 | 64.4 | 39.5 | 123.6 | 75.9 | 123.6 | 75.9 |
| 8 | Brazil | 212.30 | 54.14 | 25.5 | 158.15 | 74.5 | 0.0 | 0.0 | 5.0 | 2.3 | 10.7 | 5.0 | 25.1 | 11.8 | 0.0 | 0.0 |
| 9 | Russia | 145.60 | 8.59 | 5.9 | 137.00 | 94.1 | 0.0 | 0.0 | 0.0 | 0.0 | 0.5 | 0.3 | 4.9 | 3.4 | 0.0 | 0.0 |
| 10 | Mexico | 128.79 | 8.68 | 6.7 | 120.11 | 93.3 | 0.0 | 0.0 | 2.1 | 1.6 | 7.9 | 6.1 | 27.5 | 21.4 | 0.0 | 0.0 |
| 11 | Japan | 123.47 | 7.82 | 6.3 | 115.65 | 93.7 | 0.0 | 0.0 | 0.8 | 0.7 | 1.1 | 0.9 | 1.4 | 1.1 | 0.0 | 0.0 |
| 12 | Ethiopia | 114.83 | 0.78 | 0.7 | 114.05 | 99.3 | 0.0 | 0.0 | 27.0 | 23.5 | 68.4 | 59.6 | 100.3 | 87.3 | 0.0 | 0.0 |
| 13 | Egypt, Arab Rep. | 102.22 | 4.77 | 4.7 | 97.45 | 95.3 | 0.0 | 0.0 | 3.9 | 3.8 | 29.4 | 28.8 | 72.1 | 70.6 | 0.0 | 0.0 |
| 14 | Vietnam | 96.80 | 3.32 | 3.4 | 93.48 | 96.6 | 15.6 | 16.2 | 1.7 | 1.7 | 5.9 | 6.1 | 20.1 | 20.8 | 2.4 | 2.5 |
| 15 | Congo, Dem. Rep. | 89.41 | 0.83 | 0.9 | 88.57 | 99.1 | 11.5 | 12.9 | 65.9 | 73.7 | 80.1 | 89.6 | 86.3 | 96.6 | 10.8 | 12.1 |
| 16 | Germany | 83.70 | 0.61 | 0.7 | 83.10 | 99.3 | 0.0 | 0.0 | 0.1 | 0.1 | 0.2 | 0.2 | 0.4 | 0.5 | 0.0 | 0.0 |
| 17 | Iran, Islamic Rep. | 83.76 | 1.21 | 1.4 | 82.55 | 98.6 | 0.1 | 0.1 | 0.3 | 0.4 | 2.7 | 3.2 | 12.1 | 14.4 | 0.0 | 0.0 |
| 18 | Türkiye | 82.46 | 4.22 | 5.1 | 78.24 | 94.9 | 0.0 | 0.0 | 0.0 | 0.0 | 1.1 | 1.3 | 6.7 | 8.1 | 0.0 | 0.0 |
| 19 | Thailand | 69.68 | 2.18 | 3.1 | 67.51 | 96.9 | 0.0 | 0.0 | 0.0 | 0.0 | 0.3 | 0.5 | 5.0 | 7.2 | 0.0 | 0.0 |
| 20 | Philippines | 104.57 | 41.07 | 39.3 | 63.50 | 60.7 | 0.0 | 0.0 | 2.3 | 2.2 | 12.4 | 11.8 | 31.6 | 30.3 | 0.0 | 0.0 |
| 21 | United Kingdom | 67.23 | 4.26 | 6.3 | 62.97 | 93.7 | 0.0 | 0.0 | 0.1 | 0.2 | 0.2 | 0.3 | 0.3 | 0.5 | 0.0 | 0.0 |
| 22 | France | 64.64 | 1.74 | 2.7 | 62.90 | 97.3 | 0.0 | 0.0 | 0.0 | 0.0 | 0.0 | 0.0 | 0.0 | 0.1 | 0.0 | 0.0 |
| 23 | Italy | 60.02 | 4.13 | 6.9 | 55.89 | 93.1 | 0.0 | 0.0 | 0.8 | 1.3 | 1.0 | 1.6 | 1.7 | 2.8 | 0.0 | 0.0 |
| 24 | Tanzania | 57.89 | 4.78 | 8.3 | 53.11 | 91.7 | 0.0 | 0.0 | 26.0 | 44.9 | 40.5 | 70.0 | 48.5 | 83.7 | 0.0 | 0.0 |
| 25 | Myanmar | 53.56 | 0.90 | 1.7 | 52.66 | 98.3 | 0.2 | 0.4 | 0.5 | 0.9 | 6.4 | 11.9 | 26.0 | 48.5 | 0.1 | 0.2 |
| 26 | South Africa | 59.29 | 9.43 | 15.9 | 49.85 | 84.1 | 0.0 | 0.0 | 9.1 | 15.4 | 18.3 | 30.8 | 28.1 | 47.4 | 0.0 | 0.0 |
| 27 | Colombia | 50.78 | 1.43 | 2.8 | 49.35 | 97.2 | 1.0 | 1.9 | 1.9 | 3.7 | 5.1 | 10.1 | 13.1 | 25.8 | 0.3 | 0.6 |
| 28 | Korea, Rep. | 50.01 | 1.30 | 2.6 | 48.71 | 97.4 | 0.0 | 0.0 | 0.1 | 0.2 | 0.2 | 0.5 | 0.5 | 1.0 | 0.0 | 0.0 |
| 29 | Kenya | 53.72 | 6.30 | 11.7 | 47.42 | 88.3 | 0.0 | 0.0 | 16.2 | 30.2 | 30.3 | 56.5 | 40.7 | 75.8 | 0.0 | 0.0 |
| 30 | Uganda | 45.60 | 0.88 | 1.9 | 44.72 | 98.1 | 0.0 | 0.0 | 17.8 | 39.1 | 30.7 | 67.3 | 38.9 | 85.4 | 0.0 | 0.0 |
| 31 | Sudan | 43.72 | 0.24 | 0.6 | 43.48 | 99.4 | 0.0 | 0.0 | 5.6 | 12.7 | 19.4 | 44.4 | 34.3 | 78.4 | 0.0 | 0.0 |
| 32 | Ukraine | 43.61 | 1.02 | 2.3 | 42.59 | 97.7 | 0.0 | 0.0 | 0.0 | 0.0 | 0.2 | 0.4 | 1.5 | 3.5 | 0.0 | 0.0 |
| 33 | Algeria | 43.75 | 3.81 | 8.7 | 39.94 | 91.3 | 0.0 | 0.0 | 0.1 | 0.3 | 0.9 | 2.1 | 8.4 | 19.1 | 0.0 | 0.0 |
| 34 | Iraq | 40.15 | 0.40 | 1.0 | 39.75 | 99.0 | 0.1 | 0.2 | 0.6 | 1.4 | 5.3 | 13.2 | 19.7 | 49.1 | 0.0 | 0.1 |
| 35 | Afghanistan | 38.82 | 0.14 | 0.3 | 38.69 | 99.7 | 11.5 | 29.5 |  |  |  |  |  |  |  |  |
| 36 | Spain | 45.70 | 7.62 | 16.7 | 38.09 | 83.3 | 0.0 | 0.0 | 0.3 | 0.6 | 0.4 | 0.9 | 0.8 | 1.7 | 0.0 | 0.0 |
| 37 | Poland | 37.81 | 0.26 | 0.7 | 37.55 | 99.3 | 0.0 | 0.0 | 0.1 | 0.3 | 0.2 | 0.5 | 0.4 | 1.1 | 0.0 | 0.0 |
| 38 | Argentina | 44.74 | 10.68 | 23.9 | 34.06 | 76.1 | 0.0 | 0.0 |  |  |  |  |  |  |  |  |
| 39 | Saudi Arabia | 34.60 | 0.71 | 2.0 | 33.90 | 98.0 | 1.1 | 3.2 |  |  |  |  |  |  |  |  |
| 40 | Uzbekistan | 33.44 | 0.36 | 1.1 | 33.07 | 98.9 | 1.4 | 4.2 | 4.0 | 12.1 | 14.2 | 42.5 | 26.3 | 78.5 | 1.1 | 3.2 |
| 41 | Canada | 36.84 | 4.46 | 12.1 | 32.39 | 87.9 | 0.0 | 0.0 | 0.1 | 0.2 | 0.2 | 0.4 | 0.2 | 0.6 | 0.0 | 0.0 |
| 42 | Angola | 32.67 | 2.02 | 6.2 | 30.66 | 93.8 | 0.0 | 0.0 | 13.6 | 41.7 | 20.7 | 63.5 | 26.6 | 81.5 | 0.0 | 0.0 |
| 43 | Peru | 32.82 | 2.40 | 7.3 | 30.42 | 92.7 | 8.5 | 25.9 | 0.8 | 2.5 | 2.6 | 7.9 | 6.8 | 20.9 | 0.6 | 1.7 |
| 44 | Morocco | 36.78 | 6.47 | 17.6 | 30.31 | 82.4 | 0.0 | 0.0 | 0.2 | 0.5 | 1.6 | 4.4 | 7.5 | 20.3 | 0.0 | 0.0 |
| 45 | Ghana | 30.99 | 1.20 | 3.9 | 29.79 | 96.1 | 19.3 | 62.3 | 3.5 | 11.5 | 8.3 | 26.8 | 15.8 | 51.1 | 8.4 | 27.2 |
| 46 | Malaysia | 31.81 | 2.02 | 6.4 | 29.79 | 93.6 | 2.7 | 8.5 | 0.0 | 0.0 | 0.0 | 0.1 | 0.5 | 1.5 | 0.0 | 0.0 |
| 47 | Nepal | 29.03 | 0.15 | 0.5 | 28.87 | 99.5 | 28.0 | 96.4 | 1.7 | 5.8 | 9.7 | 33.4 | 20.3 | 70.0 | 19.6 | 67.6 |
| 48 | Yemen Rep. | 29.68 | 1.03 | 3.5 | 28.65 | 96.5 | 0.0 | 0.2 | 14.9 | 50.3 | 23.3 | 78.5 | 27.0 | 91.1 | 0.0 | 0.2 |
| 49 | Cameroon | 26.38 | 0.30 | 1.1 | 26.09 | 98.9 | 4.0 | 15.2 | 5.8 | 22.0 | 11.1 | 42.1 | 17.4 | 66.0 | 2.4 | 9.1 |
| 50 | Côte d'Ivoire | 26.37 | 0.63 | 2.4 | 25.73 | 97.6 | 7.4 | 28.0 | 6.0 | 22.6 | 13.3 | 50.4 | 20.1 | 76.1 | 4.6 | 17.3 |
| 51 | North Korea | 25.40 | 0.80 | 3.2 | 24.59 | 96.8 | 0.4 | 1.6 |  |  |  |  |  |  |  |  |
| 52 | Niger | 24.10 | 0.68 | 2.8 | 23.42 | 97.2 | 0.0 | 0.0 | 10.1 | 42.1 | 17.9 | 74.1 | 21.9 | 90.8 | 0.0 | 0.0 |
| 53 | Mozambique | 31.09 | 9.06 | 29.1 | 22.03 | 70.9 | 0.0 | 0.0 | 13.3 | 42.8 | 17.7 | 56.9 | 20.1 | 64.7 | 0.0 | 0.0 |
| 54 | Venezuela, RB | 28.06 | 6.20 | 22.1 | 21.86 | 77.9 | 0.0 | 0.0 |  |  |  |  |  |  |  |  |
| 55 | Taiwan, China | 23.68 | 1.85 | 7.8 | 21.83 | 92.2 | 0.0 | 0.0 | 0.0 | 0.0 | 0.0 | 0.0 | 0.0 | 0.0 | 0.0 | 0.0 |
| 56 | Burkina Faso | 20.86 | 0.09 | 0.4 | 20.77 | 99.6 | 0.0 | 0.0 | 6.9 | 32.9 | 14.2 | 68.3 | 18.4 | 88.3 | 0.0 | 0.0 |
| 57 | Sri Lanka | 21.16 | 1.94 | 9.2 | 19.22 | 90.8 | 0.0 | 0.0 | 0.1 | 0.7 | 1.9 | 8.9 | 7.6 | 36.0 | 0.0 | 0.0 |
| 58 | Romania | 19.21 | 0.05 | 0.3 | 19.16 | 99.7 | 0.0 | 0.0 | 0.4 | 2.3 | 0.9 | 4.9 | 2.0 | 10.2 | 0.0 | 0.0 |
| 59 | Malawi | 19.13 | 0.69 | 3.6 | 18.44 | 96.4 | 0.0 | 0.0 | 12.5 | 65.4 | 16.2 | 84.9 | 17.8 | 92.9 | 0.0 | 0.0 |
| 60 | Zambia | 18.37 | 0.18 | 1.0 | 18.18 | 99.0 | 0.0 | 0.0 | 10.6 | 57.8 | 13.6 | 74.1 | 15.9 | 86.8 | 0.0 | 0.0 |
| 61 | Mali | 20.23 | 2.19 | 10.8 | 18.04 | 89.2 | 0.0 | 0.0 | 7.7 | 38.2 | 13.3 | 65.8 | 16.7 | 82.5 | 0.0 | 0.0 |
| 62 | Kazakhstan | 18.72 | 0.94 | 5.0 | 17.78 | 95.0 | 0.0 | 0.0 | 0.0 | 0.0 | 0.1 | 0.3 | 1.0 | 5.4 | 0.0 | 0.0 |
| 63 | Guatemala | 17.89 | 0.30 | 1.7 | 17.59 | 98.3 | 0.0 | 0.0 | 1.3 | 7.0 | 3.8 | 21.0 | 7.8 | 43.8 | 0.0 | 0.0 |
| 64 | Syria | 17.30 | 0.30 | 1.7 | 17.01 | 98.3 | 0.0 | 0.0 | 7.5 | 43.6 | 13.0 | 74.9 | 15.7 | 91.0 | 0.0 | 0.0 |
| 65 | Ecuador | 17.58 | 0.59 | 3.4 | 16.99 | 96.6 | 0.0 | 0.0 | 0.5 | 3.0 | 1.5 | 8.8 | 3.9 | 22.4 | 0.0 | 0.0 |
| 66 | Cambodia | 16.62 | 0.25 | 1.5 | 16.37 | 98.5 | 0.0 | 0.0 |  |  |  |  |  |  |  |  |
| 67 | Netherlands | 16.98 | 0.65 | 3.8 | 16.33 | 96.2 | 0.0 | 0.0 | 0.0 | 0.2 | 0.0 | 0.3 | 0.1 | 0.3 | 0.0 | 0.0 |
| 68 | Chad | 16.41 | 0.23 | 1.4 | 16.18 | 98.6 | 0.0 | 0.0 | 6.4 | 39.0 | 10.9 | 66.3 | 14.1 | 85.7 | 0.0 | 0.0 |
| 69 | Chile | 18.98 | 3.63 | 19.2 | 15.34 | 80.8 | 0.0 | 0.0 | 0.0 | 0.2 | 0.1 | 0.5 | 0.5 | 2.5 | 0.0 | 0.0 |
| 70 | Zimbabwe | 14.86 | 0.12 | 0.8 | 14.74 | 99.2 | 0.0 | 0.0 | 4.6 | 30.6 | 8.6 | 57.9 | 11.7 | 78.5 | 0.0 | 0.0 |
| 71 | Guinea | 12.90 | 0.44 | 3.4 | 12.46 | 96.6 | 0.0 | 0.0 | 2.9 | 22.7 | 7.2 | 55.9 | 10.9 | 84.4 | 0.0 | 0.0 |
| 72 | Rwanda | 12.91 | 0.69 | 5.3 | 12.22 | 94.7 | 0.0 | 0.0 | 6.4 | 49.7 | 9.6 | 74.1 | 11.1 | 86.2 | 0.0 | 0.0 |
| 73 | Senegal | 16.41 | 4.56 | 27.8 | 11.84 | 72.2 | 0.0 | 0.0 | 4.1 | 25.2 | 7.7 | 46.7 | 10.3 | 62.6 | 0.0 | 0.0 |
| 74 | Bolivia | 11.65 | 0.07 | 0.6 | 11.58 | 99.4 | 0.0 | 0.0 | 0.5 | 4.3 | 1.2 | 10.0 | 2.5 | 21.4 | 0.0 | 0.0 |
| 75 | Belgium | 11.56 | 0.17 | 1.5 | 11.39 | 98.5 | 0.0 | 0.0 | 0.0 | 0.1 | 0.0 | 0.2 | 0.0 | 0.3 | 0.0 | 0.0 |
| 76 | Burundi | 11.86 | 0.68 | 5.7 | 11.18 | 94.3 | 0.0 | 0.0 | 8.9 | 74.9 | 10.4 | 87.4 | 11.0 | 92.3 | 0.0 | 0.0 |
| 77 | South Sudan | 11.18 | 0.04 | 0.3 | 11.14 | 99.7 | 0.0 | 0.0 | 9.5 | 84.8 | 10.6 | 94.8 | 11.0 | 98.7 | 0.0 | 0.0 |
| 78 | Benin | 12.06 | 0.99 | 8.2 | 11.06 | 91.8 | 6.8 | 56.3 | 5.3 | 43.9 | 8.3 | 69.2 | 10.2 | 84.5 | 6.1 | 50.8 |
| 79 | Tunisia | 11.75 | 0.96 | 8.1 | 10.80 | 91.9 | 0.0 | 0.0 | 0.0 | 0.4 | 0.5 | 4.3 | 2.6 | 22.2 | 0.0 | 0.0 |
| 80 | Somalia | 15.81 | 5.04 | 31.8 | 10.78 | 68.2 | 0.0 | 0.0 |  |  |  |  |  |  |  |  |
| 81 | Czech Republic | 10.70 | 0.04 | 0.4 | 10.66 | 99.6 | 0.0 | 0.0 | 0.0 | 0.1 | 0.0 | 0.1 | 0.0 | 0.4 | 0.0 | 0.0 |
| 82 | Jordan | 10.19 | 0.19 | 1.8 | 10.01 | 98.2 | 0.0 | 0.0 | 0.0 | 0.2 | 0.3 | 2.7 | 2.3 | 22.4 | 0.0 | 0.0 |
| 83 | Cuba | 11.25 | 1.27 | 11.3 | 9.98 | 88.7 | 0.0 | 0.0 |  |  |  |  |  |  |  |  |
| 84 | Hungary | 9.64 | 0.09 | 0.9 | 9.56 | 99.1 | 0.0 | 0.0 | 0.1 | 0.5 | 0.1 | 1.1 | 0.3 | 2.7 | 0.0 | 0.0 |
| 85 | Tajikistan | 9.53 | 0.11 | 1.2 | 9.42 | 98.8 | 0.2 | 2.6 | 0.2 | 2.3 | 1.2 | 12.2 | 3.9 | 40.8 | 0.1 | 1.1 |
| 86 | Honduras | 9.86 | 0.45 | 4.6 | 9.41 | 95.4 | 0.0 | 0.0 |  |  |  |  |  |  |  |  |
| 87 | Belarus | 9.44 | 0.14 | 1.5 | 9.30 | 98.5 | 0.0 | 0.0 | 0.0 | 0.0 | 0.0 | 0.0 | 0.0 | 0.4 | 0.0 | 0.0 |
| 88 | Austria | 8.98 | 0.05 | 0.5 | 8.94 | 99.5 | 0.0 | 0.0 | 0.0 | 0.3 | 0.0 | 0.4 | 0.1 | 0.7 | 0.0 | 0.0 |
| 89 | United Arab Emirates | 9.42 | 0.66 | 7.0 | 8.76 | 93.0 | 0.0 | 0.0 | 0.0 | 0.0 | 0.0 | 0.0 | 0.0 | 0.0 | 0.0 | 0.0 |
| 90 | Serbia | 8.74 | 0.01 | 0.2 | 8.72 | 99.8 | 0.0 | 0.0 | 0.0 | 0.0 | 0.1 | 0.7 | 0.5 | 5.7 | 0.0 | 0.0 |
| 91 | Greece | 9.94 | 1.28 | 12.9 | 8.66 | 87.1 | 0.0 | 0.0 | 0.1 | 0.8 | 0.1 | 1.4 | 0.4 | 3.9 | 0.0 | 0.0 |
| 92 | Haiti | 11.25 | 2.64 | 23.5 | 8.61 | 76.5 | 0.0 | 0.0 | 2.3 | 20.1 | 4.6 | 40.5 | 6.9 | 61.4 | 0.0 | 0.0 |
| 93 | Dominican Republic | 10.78 | 2.40 | 22.3 | 8.38 | 77.7 | 0.0 | 0.0 |  |  |  |  |  |  |  |  |
| 94 | Azerbaijan | 8.54 | 0.24 | 2.8 | 8.30 | 97.2 | 0.0 | 0.0 | 0.0 | 0.0 | 0.0 | 0.0 | 0.0 | 0.0 | 0.0 | 0.0 |
| 95 | Sweden | 9.92 | 1.66 | 16.7 | 8.27 | 83.3 | 0.0 | 0.0 | 0.0 | 0.2 | 0.0 | 0.2 | 0.0 | 0.5 | 0.0 | 0.0 |
| 96 | Switzerland | 8.61 | 0.36 | 4.2 | 8.25 | 95.8 | 0.0 | 0.0 |  |  |  |  |  |  |  |  |
| 97 | Togo | 8.13 | 0.29 | 3.6 | 7.84 | 96.4 | 5.5 | 67.4 | 3.5 | 43.3 | 5.6 | 68.5 | 7.0 | 85.6 | 4.7 | 57.6 |
| 98 | Israel | 8.34 | 1.01 | 12.1 | 7.33 | 87.9 | 0.0 | 0.0 | 0.0 | 0.2 | 0.0 | 0.6 | 0.2 | 2.2 | 0.0 | 0.0 |
| 99 | Australia | 24.72 | 17.42 | 70.5 | 7.30 | 29.5 | 0.0 | 0.0 | 0.0 | 0.2 | 0.1 | 0.2 | 0.1 | 0.2 | 0.0 | 0.0 |
| 100 | Lao PDR | 7.23 | 0.03 | 0.4 | 7.19 | 99.6 | 0.0 | 0.0 | 0.6 | 7.9 | 2.4 | 33.9 | 5.1 | 70.6 | 0.0 | 0.0 |
| 101 | Paraguay | 7.11 | 0.09 | 1.2 | 7.03 | 98.8 | 0.0 | 0.0 | 0.1 | 1.4 | 0.4 | 5.7 | 1.1 | 15.6 | 0.0 | 0.0 |
| 102 | Sierra Leone | 7.86 | 0.90 | 11.4 | 6.97 | 88.6 | 0.0 | 0.0 | 3.3 | 42.2 | 5.6 | 71.6 | 6.6 | 84.1 | 0.0 | 0.0 |
| 103 | Bulgaria | 6.89 | 0.08 | 1.2 | 6.81 | 98.8 | 0.0 | 0.0 | 0.1 | 1.3 | 0.2 | 3.0 | 0.5 | 7.3 | 0.0 | 0.0 |
| 104 | Kyrgyz Republic | 6.50 | 0.10 | 1.5 | 6.40 | 98.5 | 0.2 | 3.0 | 0.0 | 0.6 | 0.7 | 10.6 | 3.5 | 53.7 | 0.1 | 1.9 |
| 105 | El Salvador | 6.47 | 0.19 | 2.9 | 6.28 | 97.1 | 0.0 | 0.0 | 0.1 | 1.5 | 0.5 | 7.6 | 1.6 | 25.3 | 0.0 | 0.0 |
| 106 | Nicaragua | 6.62 | 0.35 | 5.3 | 6.27 | 94.7 | 0.0 | 0.0 | 0.2 | 2.8 | 0.7 | 11.2 | 2.1 | 31.5 | 0.0 | 0.0 |
| 107 | Turkmenistan | 6.02 | 0.24 | 4.1 | 5.78 | 95.9 | 0.0 | 0.0 | 0.1 | 1.0 | 0.6 | 10.1 | 1.9 | 31.9 | 0.0 | 0.0 |
| 108 | Libya | 6.84 | 1.13 | 16.5 | 5.71 | 83.5 | 0.0 | 0.0 |  |  |  |  |  |  |  |  |
| 109 | Slovak Republic | 5.45 | 0.01 | 0.3 | 5.44 | 99.7 | 0.0 | 0.0 |  |  |  |  |  |  |  |  |
| 110 | Portugal | 9.64 | 4.26 | 44.1 | 5.38 | 55.9 | 0.0 | 0.0 | 0.0 | 0.2 | 0.0 | 0.3 | 0.1 | 0.9 | 0.0 | 0.0 |
| 111 | Denmark | 5.75 | 0.44 | 7.6 | 5.31 | 92.4 | 0.0 | 0.0 |  |  |  |  |  |  |  |  |
| 112 | Congo, Rep. | 5.45 | 0.20 | 3.6 | 5.26 | 96.4 | 2.7 | 50.4 | 2.9 | 53.1 | 3.9 | 72.2 | 4.7 | 85.7 | 2.4 | 43.8 |
| 113 | Hong Kong SAR, China | 6.04 | 0.93 | 15.4 | 5.11 | 84.6 | 0.0 | 0.0 |  |  |  |  |  |  |  |  |
| 114 | Papua New Guinea | 8.83 | 3.78 | 42.8 | 5.06 | 57.2 | 0.0 | 0.0 | 1.5 | 16.5 | 2.8 | 31.5 | 4.0 | 45.4 | 0.0 | 0.0 |
| 115 | Singapore | 5.71 | 0.85 | 14.9 | 4.86 | 85.1 | 4.9 | 85.1 |  |  |  |  |  |  |  |  |
| 116 | Central African Republic | 4.79 | 0.06 | 1.2 | 4.73 | 98.8 | 0.7 | 15.6 | 3.4 | 71.7 | 4.1 | 85.8 | 4.5 | 93.7 | 0.7 | 13.6 |
| 117 | Lebanon | 6.33 | 1.66 | 26.2 | 4.67 | 73.8 | 0.0 | 0.0 | 0.0 | 0.0 | 0.0 | 0.0 | 0.1 | 1.3 | 0.0 | 0.0 |
| 118 | West Bank and Gaza | 4.92 | 0.33 | 6.7 | 4.59 | 93.3 | 0.0 | 0.0 | 0.0 | 0.8 | 0.2 | 4.1 | 1.0 | 20.0 | 0.0 | 0.0 |
| 119 | Finland | 5.52 | 1.09 | 19.7 | 4.43 | 80.3 | 0.0 | 0.0 | 0.0 | 0.1 | 0.0 | 0.1 | 0.0 | 0.1 | 0.0 | 0.0 |
| 120 | Costa Rica | 5.08 | 0.67 | 13.3 | 4.40 | 86.7 | 0.0 | 0.0 | 0.1 | 1.2 | 0.1 | 2.8 | 0.5 | 8.9 | 0.0 | 0.0 |
| 121 | Oman | 5.00 | 0.64 | 12.9 | 4.36 | 87.1 | 0.2 | 4.5 |  |  |  |  |  |  |  |  |
| 122 | Liberia | 5.03 | 0.72 | 14.3 | 4.31 | 85.7 | 0.0 | 0.0 | 2.1 | 41.8 | 3.4 | 68.3 | 4.1 | 81.2 | 0.0 | 0.0 |
| 123 | Moldova | 4.01 | 0.05 | 1.2 | 3.96 | 98.8 | 0.0 | 0.0 | 0.0 | 0.0 | 0.0 | 0.8 | 0.5 | 11.3 | 0.0 | 0.0 |
| 124 | Croatia | 4.00 | 0.27 | 6.9 | 3.73 | 93.1 | 0.0 | 0.0 | 0.0 | 0.5 | 0.0 | 1.0 | 0.1 | 3.0 | 0.0 | 0.0 |
| 125 | Georgia | 3.98 | 0.37 | 9.3 | 3.61 | 90.7 | 0.0 | 0.0 | 0.2 | 3.8 | 0.5 | 13.3 | 1.5 | 36.6 | 0.0 | 0.0 |
| 126 | Eritrea | 3.52 | 0.05 | 1.5 | 3.47 | 98.5 | 0.0 | 0.0 |  |  |  |  |  |  |  |  |
| 127 | Panama | 4.26 | 0.94 | 22.0 | 3.33 | 78.0 | 0.0 | 0.0 | 0.0 | 0.8 | 0.1 | 2.9 | 0.3 | 7.7 | 0.0 | 0.0 |
| 128 | Kosovo | 3.35 | 0.04 | 1.1 | 3.32 | 98.9 | 0.0 | 0.0 | 0.0 | 0.3 | 0.1 | 2.6 | 0.7 | 20.6 | 0.0 | 0.0 |
| 129 | Kuwait | 4.14 | 0.87 | 21.0 | 3.27 | 79.0 | 2.0 | 47.7 |  |  |  |  |  |  |  |  |
| 130 | Bosnia-Herzegovina | 3.25 | 0.02 | 0.7 | 3.23 | 99.3 | 0.0 | 0.0 | 0.0 | 0.1 | 0.0 | 0.2 | 0.1 | 2.0 | 0.0 | 0.0 |
| 131 | Armenia | 2.96 | 0.05 | 1.8 | 2.91 | 98.2 | 0.0 | 0.0 | 0.0 | 1.3 | 0.3 | 8.9 | 1.2 | 41.0 | 0.0 | 0.0 |
| 132 | Albania | 2.87 | 0.14 | 5.0 | 2.73 | 95.0 | 0.0 | 0.0 | 0.0 | 1.0 | 0.2 | 6.6 | 0.8 | 28.9 | 0.0 | 0.0 |
| 133 | Norway | 5.27 | 2.55 | 48.4 | 2.72 | 51.6 | 0.0 | 0.0 | 0.0 | 0.1 | 0.0 | 0.2 | 0.0 | 0.2 | 0.0 | 0.0 |
| 134 | Lithuania | 2.72 | 0.03 | 1.2 | 2.68 | 98.8 | 0.0 | 0.0 |  |  |  |  |  |  |  |  |
| 135 | Qatar | 2.87 | 0.29 | 10.0 | 2.58 | 90.0 | 1.7 | 58.9 |  |  |  |  |  |  |  |  |
| 136 | Jamaica | 2.89 | 0.36 | 12.5 | 2.53 | 87.5 | 0.0 | 0.0 | 0.0 | 1.6 | 0.2 | 8.2 | 0.8 | 26.2 | 0.0 | 0.0 |
| 137 | Mongolia | 3.28 | 0.86 | 26.1 | 2.42 | 73.9 | 0.0 | 0.0 | 0.0 | 0.4 | 0.1 | 3.4 | 0.6 | 18.0 | 0.0 | 0.0 |
| 138 | Botswana | 2.35 | 0.05 | 2.3 | 2.29 | 97.7 | 0.0 | 0.0 | 0.3 | 13.7 | 0.8 | 35.0 | 1.4 | 58.1 | 0.0 | 0.0 |
| 139 | Lesotho | 2.14 | 0.07 | 3.3 | 2.07 | 96.7 | 0.0 | 0.0 | 0.6 | 27.1 | 1.0 | 48.7 | 1.5 | 71.4 | 0.0 | 0.0 |
| 140 | Macedonia, FYR | 2.08 | 0.02 | 1.2 | 2.06 | 98.8 | 0.0 | 0.0 | 0.1 | 4.1 | 0.2 | 8.0 | 0.4 | 17.8 | 0.0 | 0.0 |
| 141 | Gambia, The | 2.38 | 0.34 | 14.4 | 2.04 | 85.6 | 0.0 | 0.0 | 0.2 | 8.6 | 0.8 | 32.7 | 1.5 | 62.9 | 0.0 | 0.0 |
| 142 | Slovenia | 2.06 | 0.03 | 1.3 | 2.03 | 98.7 | 0.0 | 0.0 | 0.0 | 0.0 | 0.0 | 0.0 | 0.0 | 0.1 | 0.0 | 0.0 |
| 143 | Gabon | 2.17 | 0.15 | 7.1 | 2.02 | 92.9 | 0.0 | 0.2 | 0.1 | 3.9 | 0.3 | 13.7 | 0.8 | 36.7 | 0.0 | 0.1 |
| 144 | Latvia | 1.86 | 0.07 | 4.0 | 1.79 | 96.0 | 0.0 | 0.0 | 0.0 | 0.7 | 0.0 | 1.3 | 0.1 | 3.0 | 0.0 | 0.0 |
| 145 | Guinea-Bissau | 1.85 | 0.11 | 5.9 | 1.74 | 94.1 | 0.0 | 0.0 | 1.1 | 58.8 | 1.4 | 77.5 | 1.6 | 87.2 | 0.0 | 0.0 |
| 146 | Namibia | 2.54 | 0.87 | 34.2 | 1.67 | 65.8 | 0.0 | 0.0 | 0.3 | 13.3 | 0.7 | 26.6 | 1.1 | 41.9 | 0.0 | 0.0 |
| 147 | Madagascar | 27.42 | 26.17 | 95.5 | 1.25 | 4.5 | 0.0 | 0.0 | 0.9 | 3.1 | 1.1 | 3.9 | 1.2 | 4.3 | 0.0 | 0.0 |
| 148 | Equatorial Guinea | 1.39 | 0.16 | 11.2 | 1.23 | 88.8 | 0.0 | 0.0 |  |  |  |  |  |  |  |  |
| 149 | Eswatini | 1.16 | 0.01 | 0.7 | 1.15 | 99.3 | 0.0 | 0.0 | 0.3 | 28.2 | 0.6 | 51.3 | 0.8 | 70.8 | 0.0 | 0.0 |
| 150 | Estonia | 1.32 | 0.23 | 17.4 | 1.09 | 82.6 | 0.0 | 0.0 |  |  |  |  |  |  |  |  |
| 151 | Bahrain | 1.43 | 0.38 | 26.6 | 1.05 | 73.4 | 0.7 | 50.0 |  |  |  |  |  |  |  |  |
| 152 | Timor-Leste | 1.31 | 0.26 | 20.1 | 1.04 | 80.0 | 0.0 | 0.0 | 0.2 | 16.4 | 0.7 | 51.4 | 1.0 | 73.0 | 0.0 | 0.0 |
| 153 | Cyprus | 1.14 | 0.18 | 15.9 | 0.96 | 84.1 | 0.0 | 0.0 | 0.0 | 0.0 | 0.0 | 0.0 | 0.0 | 0.0 | 0.0 | 0.0 |
| 154 | Ireland | 4.91 | 4.03 | 81.9 | 0.89 | 18.1 | 0.0 | 0.0 |  |  |  |  |  |  |  |  |
| 155 | Djibouti | 0.97 | 0.22 | 22.2 | 0.76 | 77.8 | 0.0 | 0.0 | 0.2 | 20.8 | 0.4 | 38.0 | 0.6 | 58.3 | 0.0 | 0.0 |
| 156 | Bhutan | 0.75 | 0.01 | 1.2 | 0.74 | 98.8 | 0.4 | 47.2 | 0.0 | 0.7 | 0.1 | 8.0 | 0.2 | 31.3 | 0.1 | 19.9 |
| 157 | Mauritania | 4.62 | 4.00 | 86.4 | 0.63 | 13.6 | 0.0 | 0.0 | 0.1 | 1.9 | 0.2 | 5.1 | 0.5 | 10.4 | 0.0 | 0.0 |
| 158 | Luxembourg | 0.62 | 0.01 | 1.5 | 0.61 | 98.5 | 0.0 | 0.0 |  |  |  |  |  |  |  |  |
| 159 | Montenegro | 0.62 | 0.02 | 3.8 | 0.59 | 96.2 | 0.0 | 0.0 | 0.0 | 0.7 | 0.0 | 5.7 | 0.1 | 14.0 | 0.0 | 0.0 |
| 160 | Suriname | 0.58 | 0.02 | 4.0 | 0.56 | 96.0 | 0.0 | 0.0 | 0.1 | 18.6 | 0.2 | 28.7 | 0.3 | 44.5 | 0.0 | 0.0 |
| 161 | Guyana | 0.78 | 0.22 | 28.2 | 0.56 | 71.8 | 0.0 | 0.0 | 0.0 | 3.3 | 0.1 | 8.3 | 0.1 | 18.4 | 0.0 | 0.0 |
| 162 | Belize | 0.39 | 0.02 | 5.0 | 0.37 | 95.0 | 0.0 | 0.0 | 0.0 | 11.1 | 0.1 | 23.2 | 0.2 | 44.5 | 0.0 | 0.0 |
| 163 | Brunei Darussalam | 0.43 | 0.07 | 16.7 | 0.36 | 83.3 | 0.0 | 0.0 |  |  |  |  |  |  |  |  |
| 164 | Uruguay | 3.45 | 3.13 | 90.6 | 0.32 | 9.4 | 0.0 | 0.0 |  |  |  |  |  |  |  |  |
| 165 | Malta | 0.42 | 0.11 | 25.4 | 0.32 | 74.6 | 0.0 | 0.0 | 0.0 | 0.1 | 0.0 | 0.2 | 0.0 | 0.2 | 0.0 | 0.0 |
| 166 | Macau, SAR China | 0.44 | 0.16 | 35.4 | 0.29 | 64.6 | 0.0 | 0.0 |  |  |  |  |  |  |  |  |
| 167 | São Tomé and Príncipe | 0.21 | 0.09 | 40.6 | 0.12 | 59.4 | 0.0 | 0.0 | 0.0 | 21.1 | 0.1 | 38.8 | 0.1 | 51.3 | 0.0 | 0.0 |
| 168 | Andorra | 0.08 | 0.01 | 9.2 | 0.07 | 90.8 | 0.0 | 0.0 |  |  |  |  |  |  |  |  |
| 169 | New Zealand | 2.63 | 2.57 | 97.6 | 0.06 | 2.4 | 0.0 | 0.0 |  |  |  |  |  |  |  |  |
| 170 | Isle of Man | 0.08 | 0.04 | 49.1 | 0.04 | 50.9 | 0.0 | 0.0 |  |  |  |  |  |  |  |  |
| 171 | San Marino | 0.03 | 0.00 | 1.1 | 0.03 | 98.9 | 0.0 | 0.0 |  |  |  |  |  |  |  |  |
| 172 | Liechtenstein | 0.03 | 0.01 | 19.7 | 0.03 | 80.3 | 0.0 | 0.0 |  |  |  |  |  |  |  |  |
| 173 | Cayman Islands | 0.06 | 0.06 | 94.2 | 0.00 | 5.8 | 0.0 | 0.0 |  |  |  |  |  |  |  |  |
| 174 | Puerto Rico | 2.86 | 2.86 | 100.0 | 0.00 | 0.0 | 0.0 | 0.0 |  |  |  |  |  |  |  |  |
| 175 | Trinidad and Tobago | 1.38 | 1.38 | 100.0 | 0.00 | 0.0 | 0.0 | 0.0 | 0.0 | 0.0 | 0.0 | 0.0 | 0.0 | 0.0 | 0.0 | 0.0 |
| 176 | Mauritius | 1.26 | 1.26 | 100.0 | 0.00 | 0.0 | 0.0 | 0.0 | 0.0 | 0.0 | 0.0 | 0.0 | 0.0 | 0.0 | 0.0 | 0.0 |
| 177 | Comoros | 0.82 | 0.82 | 100.0 | 0.00 | 0.0 | 0.0 | 0.0 | 0.0 | 0.0 | 0.0 | 0.0 | 0.0 | 0.0 | 0.0 | 0.0 |
| 178 | Fiji | 0.79 | 0.79 | 100.0 | 0.00 | 0.0 | 0.0 | 0.0 | 0.0 | 0.0 | 0.0 | 0.0 | 0.0 | 0.0 | 0.0 | 0.0 |
| 179 | Solomon Islands | 0.59 | 0.59 | 100.0 | 0.00 | 0.0 | 0.0 | 0.0 | 0.0 | 0.0 | 0.0 | 0.0 | 0.0 | 0.0 | 0.0 | 0.0 |
| 180 | Cabo Verde | 0.52 | 0.52 | 100.0 | 0.00 | 0.0 | 0.0 | 0.0 | 0.0 | 0.0 | 0.0 | 0.0 | 0.0 | 0.0 | 0.0 | 0.0 |
| 181 | Bahamas, The | 0.37 | 0.37 | 100.0 | 0.00 | 0.0 | 0.0 | 0.0 |  |  |  |  |  |  |  |  |
| 182 | Iceland | 0.33 | 0.33 | 100.0 | 0.00 | 0.0 | 0.0 | 0.0 | 0.0 | 0.0 | 0.0 | 0.0 | 0.0 | 0.0 | 0.0 | 0.0 |
| 183 | Barbados | 0.28 | 0.28 | 100.0 | 0.00 | 0.0 | 0.0 | 0.0 |  |  |  |  |  |  |  |  |
| 184 | Vanuatu | 0.27 | 0.27 | 100.0 | 0.00 | 0.0 | 0.0 | 0.0 | 0.0 | 0.0 | 0.0 | 0.0 | 0.0 | 0.0 | 0.0 | 0.0 |
| 185 | French Polynesia | 0.26 | 0.26 | 100.0 | 0.00 | 0.0 | 0.0 | 0.0 |  |  |  |  |  |  |  |  |
| 186 | New Caledonia | 0.26 | 0.26 | 100.0 | 0.00 | 0.0 | 0.0 | 0.0 |  |  |  |  |  |  |  |  |
| 187 | Samoa | 0.20 | 0.20 | 100.0 | 0.00 | 0.0 | 0.0 | 0.0 | 0.0 | 0.0 | 0.0 | 0.0 | 0.0 | 0.0 | 0.0 | 0.0 |
| 188 | St. Lucia | 0.18 | 0.18 | 100.0 | 0.00 | 0.0 | 0.0 | 0.0 | 0.0 | 0.0 | 0.0 | 0.0 | 0.0 | 0.0 | 0.0 | 0.0 |
| 189 | Guam | 0.17 | 0.17 | 100.0 | 0.00 | 0.0 | 0.0 | 0.0 |  |  |  |  |  |  |  |  |
| 190 | Curaçao | 0.16 | 0.16 | 100.0 | 0.00 | 0.0 | 0.0 | 0.0 |  |  |  |  |  |  |  |  |
| 191 | Micronesia, Fed. Sts. | 0.11 | 0.11 | 100.0 | 0.00 | 0.0 | 0.0 | 0.0 | 0.0 | 0.0 | 0.0 | 0.0 | 0.0 | 0.0 | 0.0 | 0.0 |
| 192 | Grenada | 0.11 | 0.11 | 100.0 | 0.00 | 0.0 | 0.0 | 0.0 |  |  |  |  |  |  |  |  |
| 193 | Aruba | 0.11 | 0.11 | 100.0 | 0.00 | 0.0 | 0.0 | 0.0 |  |  |  |  |  |  |  |  |
| 194 | St. Vincent and the Grenadines | 0.10 | 0.10 | 100.0 | 0.00 | 0.0 | 0.0 | 0.0 |  |  |  |  |  |  |  |  |
| 195 | Tonga | 0.10 | 0.10 | 100.0 | 0.00 | 0.0 | 0.0 | 0.0 | 0.0 | 0.0 | 0.0 | 0.0 | 0.0 | 0.0 | 0.0 | 0.0 |
| 196 | Antigua and Barbuda | 0.09 | 0.09 | 100.0 | 0.00 | 0.0 | 0.0 | 0.0 |  |  |  |  |  |  |  |  |
| 197 | Seychelles | 0.09 | 0.09 | 100.0 | 0.00 | 0.0 | 0.0 | 0.0 | 0.0 | 0.0 | 0.0 | 0.0 | 0.0 | 0.0 | 0.0 | 0.0 |
| 198 | Dominica | 0.07 | 0.07 | 100.0 | 0.00 | 0.0 | 0.0 | 0.0 |  |  |  |  |  |  |  |  |
| 199 | Northern Mariana Islands | 0.06 | 0.06 | 100.0 | 0.00 | 0.0 | 0.0 | 0.0 |  |  |  |  |  |  |  |  |
| 200 | American Samoa | 0.05 | 0.05 | 100.0 | 0.00 | 0.0 | 0.0 | 0.0 |  |  |  |  |  |  |  |  |
| 201 | St. Kitts and Nevis | 0.05 | 0.05 | 100.0 | 0.00 | 0.0 | 0.0 | 0.0 |  |  |  |  |  |  |  |  |
| 202 | Faeroe Islands | 0.05 | 0.05 | 100.0 | 0.00 | 0.0 | 0.0 | 0.0 |  |  |  |  |  |  |  |  |
| 203 | Bermuda | 0.04 | 0.04 | 100.0 | 0.00 | 0.0 | 0.0 | 0.0 |  |  |  |  |  |  |  |  |
| 204 | Sint Maarten | 0.04 | 0.04 | 100.0 | 0.00 | 0.0 | 0.0 | 0.0 |  |  |  |  |  |  |  |  |
| 205 | Greenland | 0.04 | 0.04 | 100.0 | 0.00 | 0.0 | 0.0 | 0.0 |  |  |  |  |  |  |  |  |
| 206 | Turks and Caicos Islands | 0.04 | 0.04 | 100.0 | 0.00 | 0.0 | 0.0 | 0.0 |  |  |  |  |  |  |  |  |
| 207 | Marshall Islands | 0.04 | 0.04 | 100.0 | 0.00 | 0.0 | 0.0 | 0.0 |  |  |  |  |  |  |  |  |
| 208 | Saint-Martin | 0.03 | 0.03 | 100.0 | 0.00 | 0.0 | 0.0 | 0.0 |  |  |  |  |  |  |  |  |
| 209 | Maldives | 0.02 | 0.02 | 100.0 | 0.00 | 0.0 | 0.0 | 0.0 | 0.0 | 0.0 | 0.0 | 0.0 | 0.0 | 0.0 | 0.0 | 0.0 |
| 210 | Palau | 0.02 | 0.02 | 100.0 | 0.00 | 0.0 | 0.0 | 0.0 |  |  |  |  |  |  |  |  |
| 211 | Tuvalu | 0.01 | 0.01 | 100.0 | 0.00 | 0.0 | 0.0 | 0.0 | 0.0 | 0.0 | 0.0 | 0.0 | 0.0 | 0.0 | 0.0 | 0.0 |

*Note: Empty cells reflect the lack of input data.*

# 2. Additional figures illustrating countries’ exposure to air pollution

**Supplementary** **Figure 2.1. Absolute population exposure to high concentrations of air pollution at the country level (millions)**
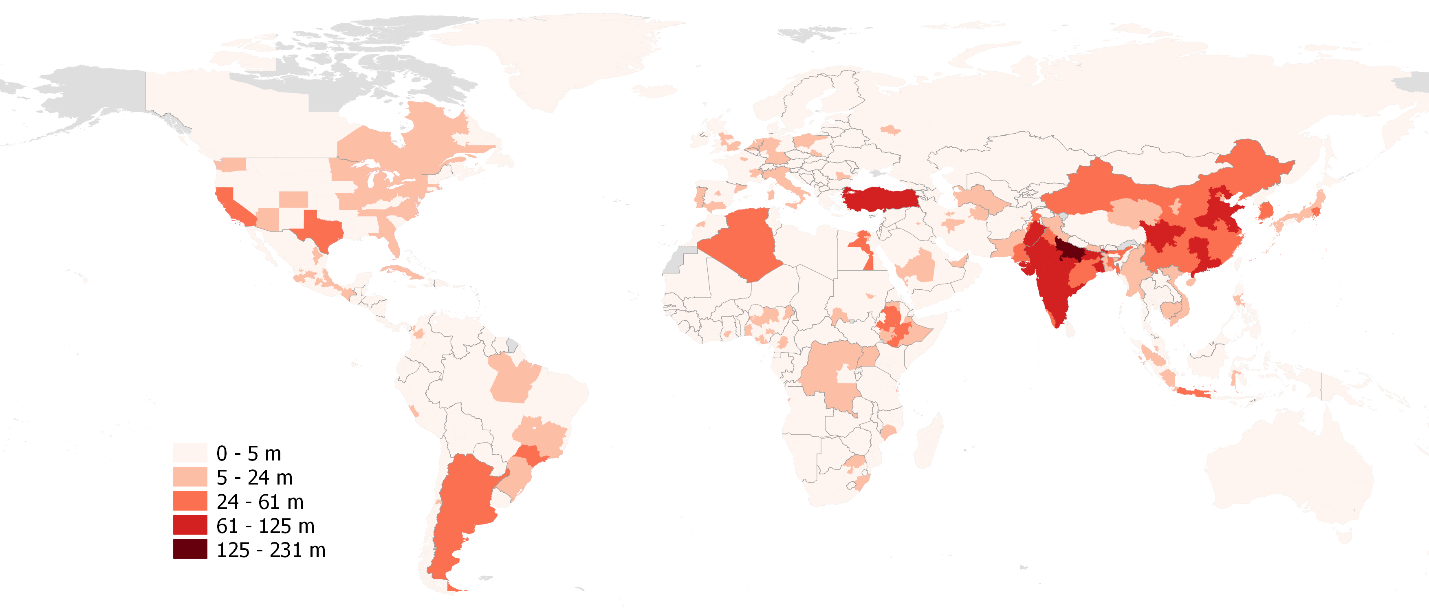


**Supplementary** **Figure 2.2. Top 10 countries: share of population exposed to hazardous concentrations of air pollution**


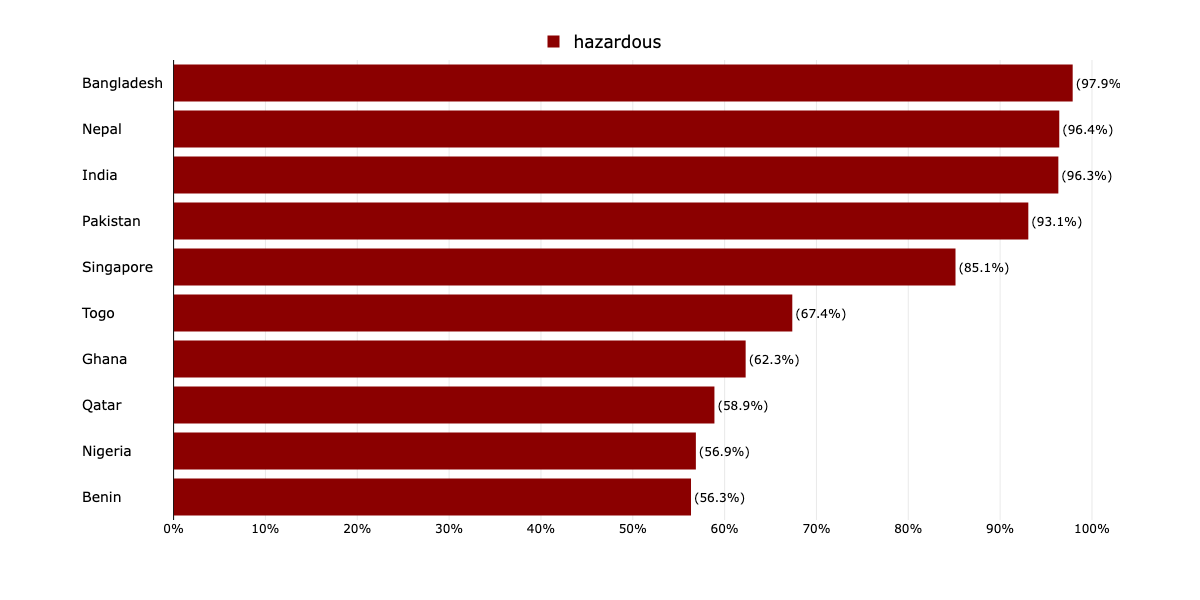


**Supplementary** **Figure 2.3. Top 10 countries: number of people living on less than $1.90 a day exposed to unsafe PM2.5 levels**


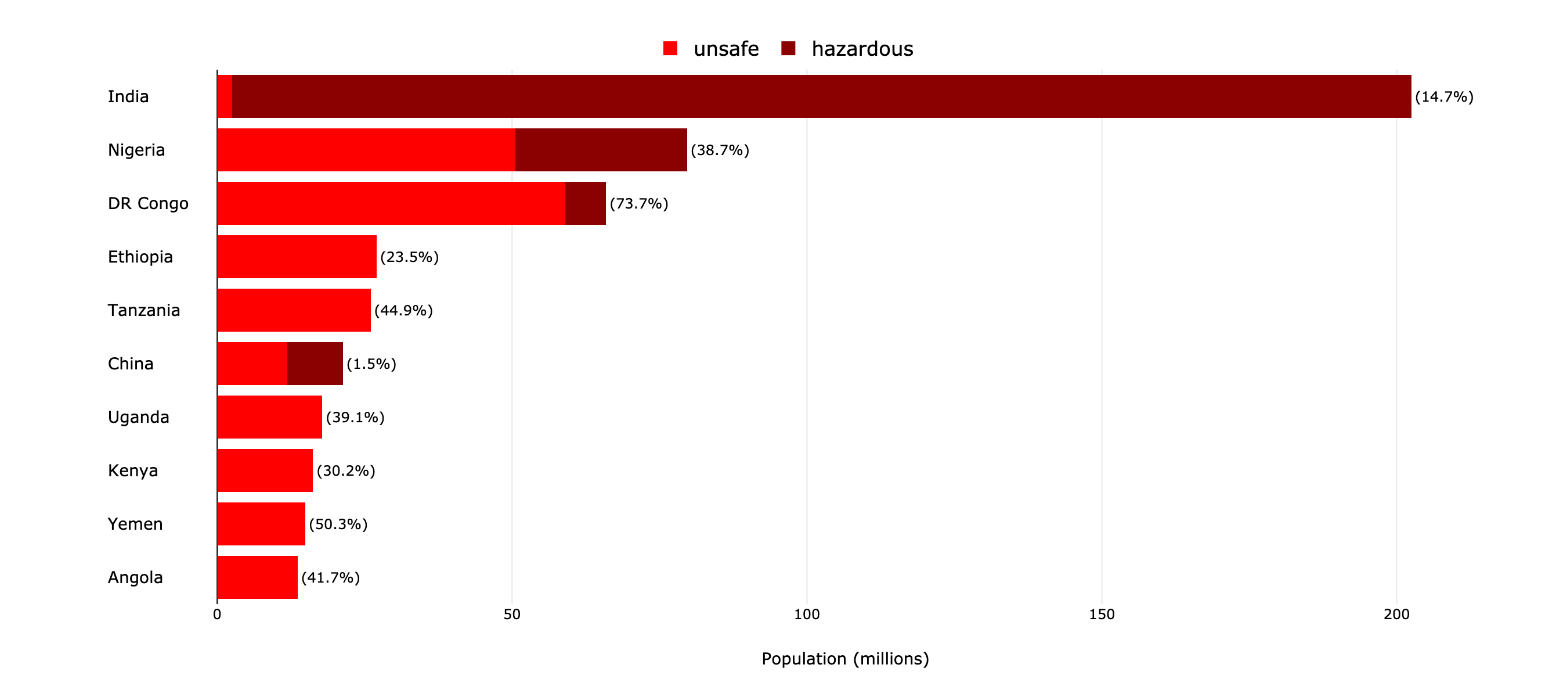


# 3. The relationship between income and air pollution levels

To assess the relationship between pollution intensities and income levels, it is useful to consider the full range of PM2.5 concentrations. Computing spatially averaged PM2.5 concentrations for each of the 2,183 subnational areas in this study and comparing these with population and income data suggests that areas with larger populations tend to have higher pollution levels, and average pollution levels appear particularly high for areas in the middle-income category (Supplementary figure 3.1).

To further examine this relationship statistically, we consider the full sample of subnational areas and a subset of high-pollution areas with average concentrations over 10μg/m^3^ (Supplementary table 3.1). The role of population size is consistent across all specifications. Including population as an explanatory variable for pollution levels raises explanatory power and confirms that larger populations are associated with higher pollution. This is also due to the increased polluting activities that correlate with larger populations, such as transport, electricity generation, or industrial production.

**Supplementary** **Table 3.1. The role of income and population in explaining mean PM2.5 concentration levels**

| **Mean PM2.5 concentration** | Full sample of subnational areas | | | |  | Subset of high-pollution areas with average concentrations over >10μg/m3 | | | |
| --- | --- | --- | --- | --- | --- | --- | --- | --- | --- |
|  | (I) | (II) | (III) | (IV) |  | (V) | (VI) | (VII) | (VIII) |
| Log income | -3.2670*** | 0.3342 | 2.3043** | -0.7977 |  | -3.5551*** | 2.3383 | 3.8596*** | 1.1722 |
|  | *(0.281)* | *(1.050)* | *(0.997)* | *(1.060)* |  | *(0.408)* | *(1.493)* | *(1.402)* | *(1.429)* |
| Log income^2^ |  | -0.8223*** | -1.4078*** | -0.4958** |  |  | -1.4683*** | -1.9755*** | -0.9029*** |
|  |  | *(0.231)* | *(0.221)* | *(0.231)* |  |  | *(0.358)* | *(0.337)* | *(0.309)* |
| Log population | |  | 2.7994*** | 2.3827*** |  |  |  | 3.0789*** | 2.6146*** |
|  |  |  | *(0.188)* | *(0.160)* |  |  |  | *(0.250)* | *(0.211)* |
| Constant | 22.3411*** | 19.3178*** | -20.4817*** | -9.6542*** |  | 28.0406*** | 23.5092*** | -20.2021*** | -10.4571*** |
|  | *(0.658)* | *(1.073)* | *(2.851)* | *(2.302)* |  | *(0.876)* | *(1.406)* | *(3.784)* | *(2.904)* |
| Region |  |  |  | YES |  |  |  |  | YES |
| N | 1,707 | 1,707 | 1,707 | 1,707 |  | 1,049 | 1,049 | 1,049 | 1,049 |
| adj. R2 | 0.073 | 0.079 | 0.185 | 0.420 |  | 0.067 | 0.081 | 0.196 | 0.440 |

Income levels also have some explanatory power. When focusing on areas with unsafe PM2.5 levels (here with average PM2.5 concentration over 10μg/m^3^; models V to VIII), an inverted U-type relationship is discernable with negative coefficients for squared income terms (Supplementary figure 3.2). In other words, among regions with unsafe air, PM2.5 levels increase along with income up to a level of about $9.5/day (or $3,478/year) after which they decrease with income. However, this threshold is low, meaning that, for most of the income distribution, the negative relationship dominates. Note the switching of signs on first order polynomials between the full and restricted sample. Also note the limited increase in explanatory power (R^2^) from the inclusion of squared income terms. Polynomial regression specifications can, in principle, be prone to over-fitting, but a non-parametric kernel regression (Supplementary figure 3.3) confirms the qualitative nature of the relationship between pollution and income.

**Supplementary** **Figure 3.1. Annual average PM2.5 concentrations in 2,183 subnational regions**

1. **By population b) By mean income**


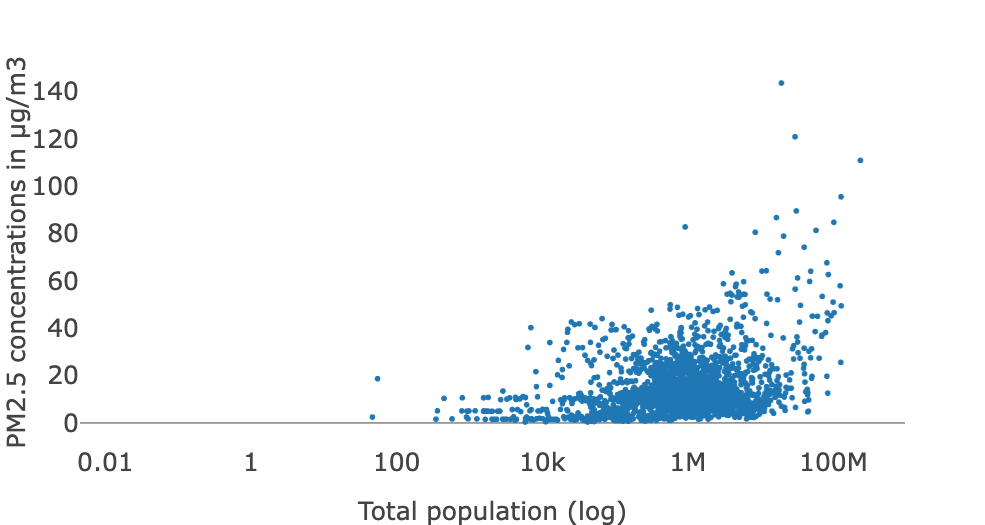

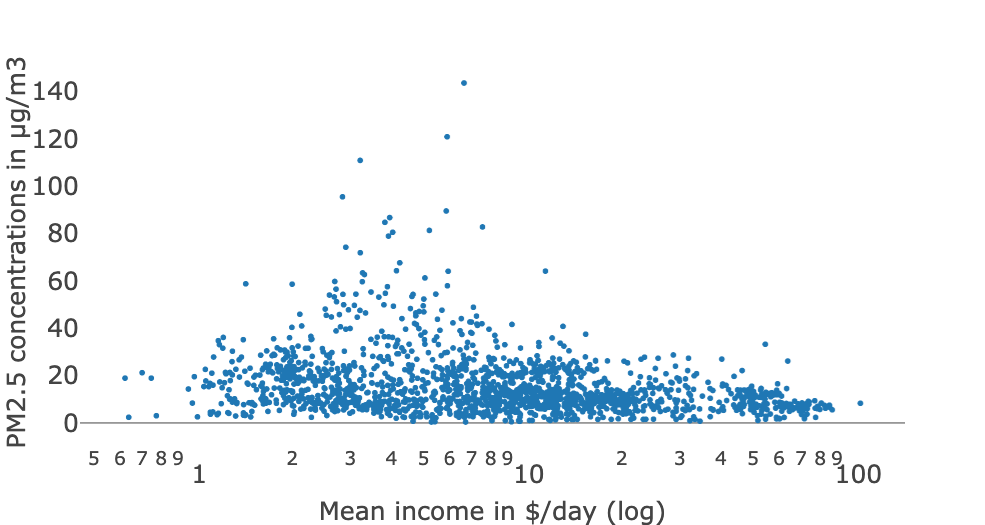


**Supplementary** **Figure 3.2. Fitted lines for the full sample of subnational areas**

| *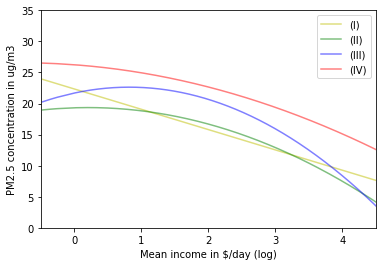* |  |
| --- | --- |

**Supplementary** **Figure 3.3. Non-parametric kernel regression results**

| 1. **Full sample** | 1. **Average concentration over >10μg/m^3^** |
| --- | --- |
| 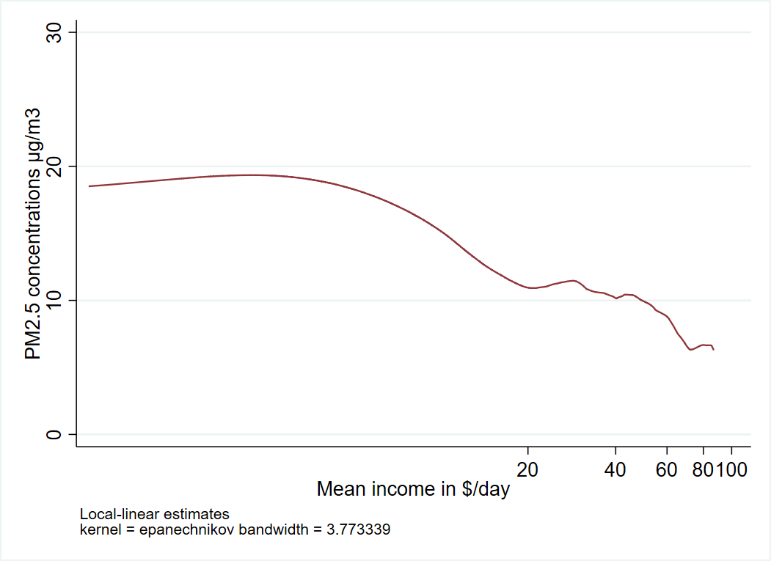 | 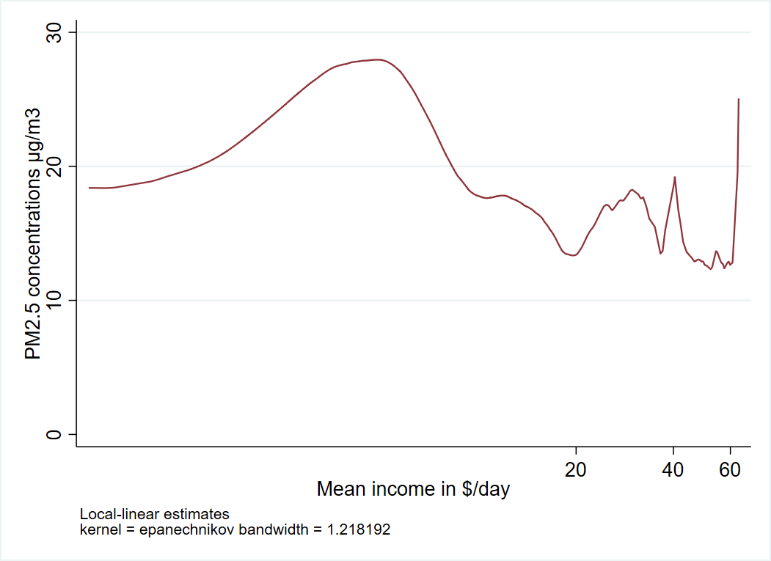 |
