# Supplementary material for: Global air pollution exposure and poverty
Source: Nat Commun. 2023 Jul 22;14:4432. doi: 10.1038/s41467-023-39797-4 (PMC10363163; doi:10.1038/s41467-023-39797-4)
Supplement: Supplementary file 3 — Reporting Summary [file 41467_2023_39797_MOESM3_ESM.pdf]

nature portfolio

Corresponding author(s): Jun Rentschler
NCOMMS-22-50739A

Last updated by author(s): Jun 12, 2023

# Reporting Summary

Nature Portfolio wishes to improve the reproducibility of the work that we publish. This form provides structure for consistency and transparency in reporting. For further information on Nature Portfolio policies, see our Editorial Policies and the Editorial Policy Checklist.

## Statistics

For all statistical analyses, confirm that the following items are present in the figure legend, table legend, main text, or Methods section.

| n/a | Confirmed | |
|---|---|---|
| ☒ | ☐ | The exact sample size ($n$) for each experimental group/condition, given as a discrete number and unit of measurement |
| ☒ | ☐ | A statement on whether measurements were taken from distinct samples or whether the same sample was measured repeatedly |
| ☒ | ☐ | The statistical test(s) used AND whether they are one- or two-sided<br>*Only common tests should be described solely by name; describe more complex techniques in the Methods section.* |
| ☒ | ☐ | A description of all covariates tested |
| ☒ | ☐ | A description of any assumptions or corrections, such as tests of normality and adjustment for multiple comparisons |
| ☐ | ☒ | A full description of the statistical parameters including central tendency (e.g. means) or other basic estimates (e.g. regression coefficient) AND variation (e.g. standard deviation) or associated estimates of uncertainty (e.g. confidence intervals) |
| ☒ | ☐ | For null hypothesis testing, the test statistic (e.g. $F$, $t$, $r$) with confidence intervals, effect sizes, degrees of freedom and $P$ value noted<br>*Give P values as exact values whenever suitable.* |
| ☒ | ☐ | For Bayesian analysis, information on the choice of priors and Markov chain Monte Carlo settings |
| ☒ | ☐ | For hierarchical and complex designs, identification of the appropriate level for tests and full reporting of outcomes |
| ☒ | ☐ | Estimates of effect sizes (e.g. Cohen's $d$, Pearson's $r$), indicating how they were calculated |

*Our web collection on statistics for biologists contains articles on many of the points above.*

## Software and code

Policy information about availability of computer code

| | |
|---|---|
| Data collection | No original primary data was collected for this study |
| Data analysis | All analysis was conducted using Python. If accepted for publication, the code will be made publicly available via GitHub. |

For manuscripts utilizing custom algorithms or software that are central to the research but not yet described in published literature, software must be made available to editors and reviewers. We strongly encourage code deposition in a community repository (e.g. GitHub). See the Nature Portfolio guidelines for submitting code & software for further information.

## Data

Policy information about availability of data

All manuscripts must include a data availability statement. This statement should provide the following information, where applicable:

- Accession codes, unique identifiers, or web links for publicly available datasets
- A description of any restrictions on data availability
- For clinical datasets or third party data, please ensure that the statement adheres to our policy

Population density data are publicly available from WorldPop at https://hub.worldpop.org/geodata/listing?id=69
PM2.5 particle concentration data are publicly available from the Atmospheric Composition Analysis Group at https://sites.wustl.edu/acag/datasets/surface-pm2-5/
Global subnational poverty data are publicly available from the World Bank at https://datacatalog.worldbank.org/search/dataset/0042041/international-poverty-line---subnational-poverty

# Human research participants

Policy information about studies involving human research participants and Sex and Gender in Research.

| | |
|---|---|
| Reporting on sex and gender | n.a. |
| Population characteristics | n.a. |
| Recruitment | n.a. |
| Ethics oversight | n.a. |

Note that full information on the approval of the study protocol must also be provided in the manuscript.

# Field-specific reporting

Please select the one below that is the best fit for your research. If you are not sure, read the appropriate sections before making your selection.

☐ Life sciences ☐ Behavioural & social sciences ☒ Ecological, evolutionary & environmental sciences

For a reference copy of the document with all sections, see nature.com/documents/nr-reporting-summary-flat.pdf

# Ecological, evolutionary & environmental sciences study design

All studies must disclose on these points even when the disclosure is negative.

| | |
|---|---|
| Study description | This study offers a comprehensive account of the relationship between ambient air pollution exposure and poverty in 211 countries and territories. This study offers the first global estimates (i) of PM2.5 exposure and its interaction with national and subnational poverty levels, and (ii) of population PM2.5 exposure, based on the WHO's revised air pollution guidelines , with detailed national and subnational estimates. It uses a spatial overlay approach of two gridded high resolution datasets (PM2.5 and population counts), and combines this with administrative unit level poverty estimates. |
| Research sample | We use global population density data for all countries, which represents all of the global population. We rely on remote sensing data collected by satellite sensors. This choice is motivated by the objective to achieve broad global coverage, representative of the global population. This dataset is the WorldPop population count data and the PM2.5 particle concentration data (sources are provided aboce in the "data availability" section). |
| Sampling strategy | n.a. (no sampling was conducted. Instead the dataset used in this study reflects the entire world population) |
| Data collection | No original primary data was collected as part of this study. |
| Timing and spatial scale | The analysis represents a 2019 snapshot of global population, poverty, and air pollution. It covers 211 countries, i.e. virtually 100% of the world population, at both national and admin1 subnational scales. |
| Data exclusions | Populations located in some politically disputed areas were excluded from the analysis, as there are no reliable administrative level data available (incl. poverty estimates). |
| Reproducibility | No experiments were conducted for this study. |
| Randomization | No experiments were conducted for this study. Hence there were no study participants, sample groups, or randomization. |
| Blinding | Blinding was not applicable. The study uses global population density maps that make individual identification impossible. |

Did the study involve field work? ☐ Yes ☒ No

# Reporting for specific materials, systems and methods

We require information from authors about some types of materials, experimental systems and methods used in many studies. Here, indicate whether each material, system or method listed is relevant to your study. If you are not sure if a list item applies to your research, read the appropriate section before selecting a response.

## Materials & experimental systems

| n/a | Involved in the study |
|---|---|
| ☒ | ☐ Antibodies |
| ☒ | ☐ Eukaryotic cell lines |
| ☒ | ☐ Palaeontology and archaeology |
| ☒ | ☐ Animals and other organisms |
| ☒ | ☐ Clinical data |
| ☒ | ☐ Dual use research of concern |

## Methods

| n/a | Involved in the study |
|---|---|
| ☒ | ☐ ChIP-seq |
| ☒ | ☐ Flow cytometry |
| ☒ | ☐ MRI-based neuroimaging |
